# The effects of aerobic and resistance exercise on the lipid profile of extracellular vesicles

**DOI:** 10.1007/s00421-025-05973-1

**Published:** 2025-10-01

**Authors:** Tesha Kerr Carpenter, Mariah McCashland, Raechel Sherrick, Sathish Kumar Natarajan, Mirko Mandić, Stephen D. Kachman, Ferdinand von Walden, Ivan J. Vechetti, Rodrigo Fernandez-Gonzalo

**Affiliations:** 1 Department of Nutrition and Health Sciences, University of Nebraska-Lincoln, Lincoln, NE USA; 2 Department of Laboratory Medicine, Division of Clinical Physiology, Karolinska Institutet, Stockholm, Sweden; 3 Unit of Clinical Physiology, Karolinska University Hospital, Stockholm, Sweden; 4 Department of Statistics, University of Nebraska-Lincoln, Lincoln, NE USA; 5 Department of Women’s and Children’s Health, Karolinska Institutet, Solna, Sweden

**Keywords:** Extracellular vesicle, Resistance exercise, Aerobic exercise, Lipidomics

## Abstract

**Purpose:**

Lipids are one of the most abundant molecules within extracellular vesicles (EVs) and are important for EV biology and cell signaling. However, very little attention has been given to the role of lipids in the biological function of EVs during exercise. Therefore, we completed a study using a cross-over design to investigate the effects of aerobic and resistance exercise on the lipid profile of EVs.

**Methods:**

Ten healthy participants (23.0 ± 3.6 years) performed an acute bout of aerobic exercise, an acute bout of resistance exercise, as well as a period of rest (control) in a randomized, cross-over design. Blood samples were collected immediately following exercise and 30 min after exercise, and exercise conditions were compared to control within each subject. Plasma EVs were isolated using cushioned-density gradient ultracentrifugation (C-DGUC). The EV size, morphology, and protein markers were examined using nanoflow cytometry, transmission electron microscopy, and western blot, respectively. Additionally, we conducted an untargeted lipidomics analysis on the EV isolate.

**Results:**

Our findings revealed neither exercise modality had a significant effect on the size or concentration of EVs (*P* > 0.05). However, we found that immediately after exercise there was a decrease in glycerophospholipids within the EVs (55% vs 49%; *P* < 0.05) in the resistance exercise group compared to the control group, a response not found in the aerobic exercise group.

**Conclusion:**

Our data suggest that although resistance exercise induced some changes in the lipid composition of EVs, the EV-containing lipids do not appear to be a critical mechanism utilized by cells to mediate exercise-induced adaptations.

**Graphical Abstract:**

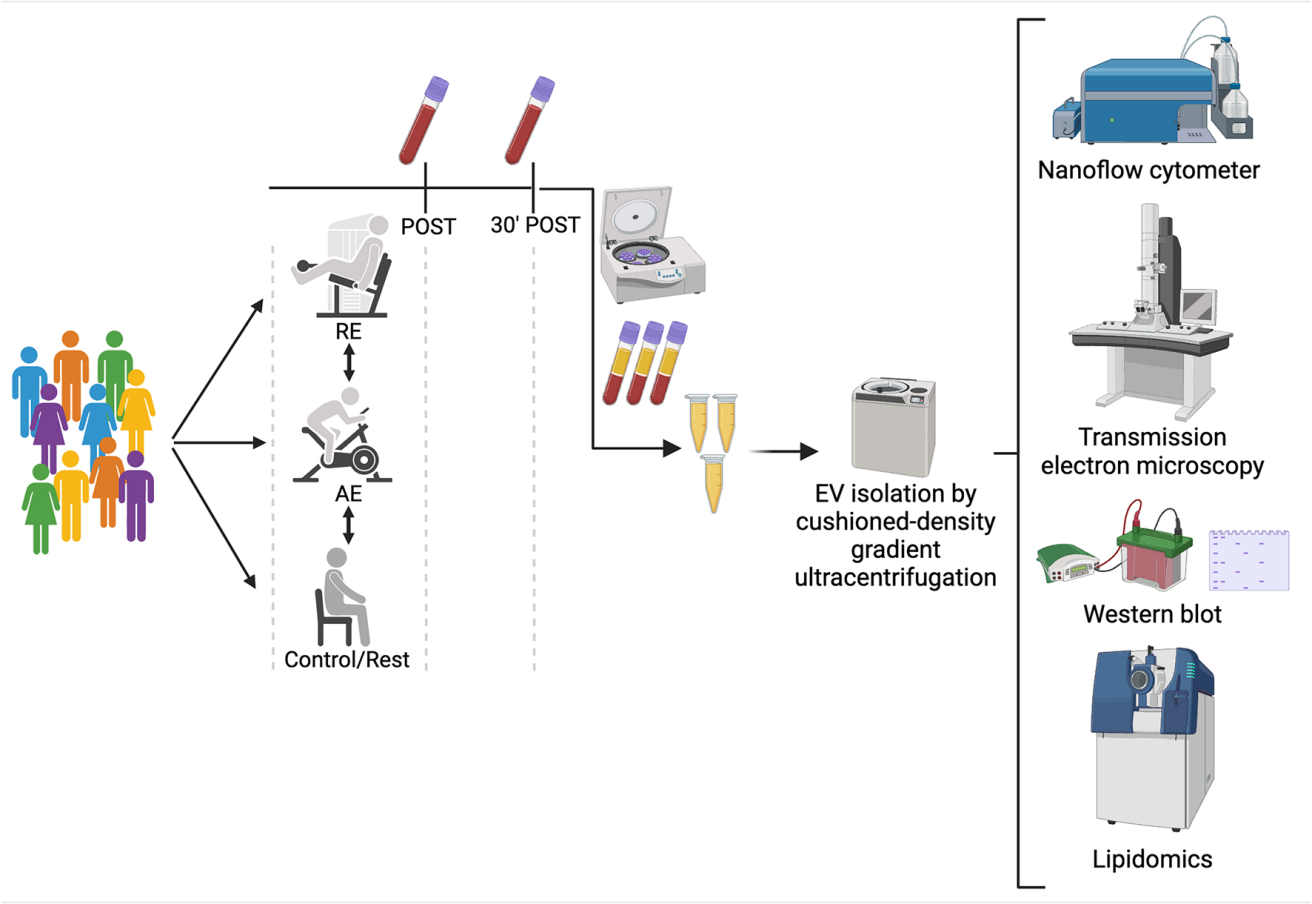

**Supplementary Information:**

The online version contains supplementary material available at 10.1007/s00421-025-05973-1.

## Introduction

Extracellular vesicles (EVs) are membrane-bound vesicles that are released in a conserved manner by virtually all cells in the body. Extracellular vesicles are biologically active and carry a heterogeneous cargo of bioactive molecules, including nucleic acids, proteins, sugars, and lipids, all of which have the potential to alter the function of the recipient cell (Raposo et al. 1996; Ratajczak et al. 2006; Valadi et al. 2007) and suggest EVs as cornerstones of intercellular communication.

It is important to note that while the term EV is commonly used to refer to secreted membrane vesicles, the actual population of secreted vesicles is very diverse. Classification of these vesicles is largely based on the process of biogenesis. For example, exosomes and ectosomes differ with respect to their site of production, with exosomes forming at the limiting membrane of multivesicular endosomes and ectosomes forming at the plasma membrane (Mathieu et al. 2021; Dixson et al. 2023). However, current isolation methods do not allow for the separation of vesicles based on their site of biogenesis, making it difficult to obtain pure populations of either exosomes or ectosomes. Additionally, there are still controversies regarding the differences between exosomes and ectosomes (Fordjour et al. 2022). Therefore, in this study and in accordance with the recommendations of the International Society of Extracellular Vesicles, we will refer to our isolates as EVs (Lötvall et al. 2014; Théry et al. 2018; Welsh et al. 2024).

Physical activity is well known for its myriad of positive effects on human health, including a reduced risk of various chronic diseases, increased quality of life, and increased longevity in certain contexts (Warburton et al. 2006; Pedersen and Fischer 2007; Pedersen 2009; Woodcock et al. 2011; Booth et al. 2012). Despite the well-established benefits, the precise mechanisms by which tissues not directly involved in exercise are affected are not well understood. The prevailing hypothesis for these benefits is the release of exercise factors into the circulation, which facilitates the multisystemic benefits and counteracts the adverse effects of a sedentary lifestyle (Steensberg et al. 2000; Pedersen and Fischer 2007; Sigal et al. 2014). Multiple cell types release factors during exercise (Lee et al. 2019) and it is currently believed that many of these exercise factors are packaged and released as EV cargo (Wubbolts et al. 2003; Valadi et al. 2007; Montecalvo et al. 2012; Cheng et al. 2014; Pienimaeki-Roemer et al. 2015).

The majority of studies examining the relationship between EVs and exercise have primarily focused on miRNAs (Fry et al. 2017; Yin et al. 2019; de Mendonça et al. 2020; Castaño et al. 2020; Di et al. 2020; Vechetti et al. 2021), although there is evidence suggesting that miRNAs are minor constituents of EVs that are rarely delivered to target cells (Chevillet et al. 2014; Albanese et al. 2021). If this mechanism is conserved across cell types and physiological/pathophysiological conditions, studies demonstrating the effects of miRNAs delivered by EVs are likely a synergistic effect of additional EV cargo besides miRNAs. Indeed, different EV cargoes have been shown to impact exercise-mediated changes. For example, protein-containing EVs are transferred to other cells and have an effect on metabolism and influence aging and senescence (Whitham et al. 2018; McIlvenna and Whitham 2023). Furthermore, we have demonstrated that during a hypertrophic stimulus, muscle-derived EVs containing miR-1 stimulate adipose tissue lipolysis (Vechetti et al. 2021). In addition to miRNAs and proteins, lipids, one of the most abundant molecules within EVs, are essential for EV biology and potent signaling molecules. As of yet, very little attention has been given to the role of lipids in the biological function of EVs. Lipids play an essential role in EV biogenesis and it has been found that the biogenesis of exosomes is promoted by the conversion of sphingomyelin to ceramide (Trajkovic et al. 2008). Furthermore, lipid cargoes such as arachidonic acid (AA) and polyunsaturated fatty acids (PUFAs) provide a pool of molecules that can be modified into signaling molecules such as eicosanoids that play a role in a variety of biological functions and disease states (Harizi et al. 2008; Fyfe et al. 2023) In addition, we have recently demonstrated that the lipid composition of muscle cell-derived EVs is altered during a hypertrophic stimulus in vitro (Valentino et al. 2021). Despite these changes, to our knowledge, no other study has investigated the role of lipids within EVs during exercise. Moreover, studies investigating the effects of different exercise modalities on EV concentration and size are limited and conflicting, likely due to inconsistencies in methodologies used (exercise length and intensity, blood collection and procedures, methods of EV isolation and characterization, and others).

Therefore, the primary objective of our study was to examine the impact of different exercise modalities (i.e., aerobic and resistance) on the concentration, size, and lipid content of EVs released immediately following and 30 min after an acute bout of physical exercise. We hypothesized that exercise would have an effect on the concentration, size, and lipid species of EVs, and that these changes would be exercise type-specific. Contrary to our hypothesis, we did not observe changes in EV concentration, regardless of the type of exercise or time point post-exercise, and only observed minor changes in the lipid profile of EVs following resistance but not aerobic exercise.

## Materials and methods

### General design

Ten individuals (five men and five women) engaged in acute sessions of aerobic exercise, resistance exercise, or rest/control in a randomized cross-over design (Table 1). The use of a cross-over design allows for intrasubject comparisons, and thus the control of e.g., genetic factors and physical activity experience, which reduces the variability in the responses to an intervention and in turn the number of participants required. Blood samples were collected immediately after and 30 min following a bout of exercise or a resting period. Extracellular vesicles were isolated from plasma and analyzed for size, concentration, protein markers, and lipidomics.

**Table 1:** Descriptive characteristics, maximal voluntary isometric contraction (MVIC) and cardiopulmonary exercise test result in the 10 participants (5 men and 5 women)

	Age (year)	Weight (kg)	Height (cm)	MVIC (kg)	*V̇*O_2max_ (ml/kg/min)	HR_max_ (bpm)	*W*_max_
Mean	26.0	72.4	172.6	135.4	39.3	183.0	252.2
SD	3.6	15.0	11.1	43.4	5.9	11.8	60.7
Minimum	20.8	51.0	157.5	84.0	32.3	160.0	164.0
Maximum	30.6	101.6	190.0	200.0	47.4	199.0	337.0

*V̇*O_2max_ Maximal oxygen uptake, *W*_max_ Maximal workload during the maximal incremental test, *HR*_max_ Maximum heart rate

### Subjects

Ten moderately active men and women (18–35 years old) with moderate prior experience in aerobic and resistance exercise were recruited from the greater Stockholm area using social media and institutional recruitment websites. The recruitment period started on February 1, 2021, and concluded on April 30, 2021. Exclusion criteria were contraindications to strenuous exercise and any chronic health conditions. The number of participants was calculated considering potential differences in EV concentration based on previous research using a similar type of resistance exercise bout (Annibalini et al. 2019). The study experiments and procedures were explained before subjects gave their written informed consent to participate. The study was approved by the Swedish Ethical Review Authority (#2020-06467) and was conducted in accordance with the Declaration of Helsinki (except for registration in a database).

### Familiarization, baseline testing, and exercise bouts

Subjects reported to the laboratory four times, with at least 1 week between sessions. The first visit included lower limb maximal isometric strength, an incremental *V̇*O_2max_ test, and familiarization with the resistance exercise equipment. Isometric strength was measured standing on a dynamometer platform with flexed knees, straight arms, and a straight back at baseline (i.e., squat position), and holding a handle connected to the dynamometer. The study participants had an isometric squat force of 135.4 ± 43.4 kg (Table 1). To determine *V̇*O_2max_ and maximal workload (*W*_max_), subjects performed an incremental cycling test to volitional fatigue on an electronically braked ergometer (Monark LC6, Vansbro, Sweden). Using an online gas collection system (COSMED Quark CPET, Rome, Italy) fractions of inspired and expired O_2_ and CO_2_ were measured continuously and recorded as breath-by-breath values. Subjects began the test at 50W for 5 min as a warm-up. Subsequently, resistance was increased by 1W every 3 s, corresponding to an increase of 20W per minute, until subjects reached volitional fatigue. *V̇*O_2max_ was considered to be the highest 20-s average value achieved during the test. Criteria for the test to be considered maximal were either a plateau in *V̇*O_2_ or fulfillment of the following; RER > 1.15, a heart rate within 10 beats of age-predicted maximum, and volitional exhaustion. On average, participants in the current study showed *V̇*O_2max_ values of 39.3 ± 5.9 ml/kg/min (Table 1). The familiarization with the resistance exercise equipment consisted of 4 sets of 7 sub-maximal squats using an iso-inertial flywheel ergometer (Exxentric, Bromma, Sweden) and 4 sets of 7 sub-maximal repetitions using an iso-inertial knee extension device (YoYo™ Technology AB, Stockholm, Sweden). When performed correctly, isoinertial resistance exercise technology promotes overload during the eccentric part of the action, which is considered important for triggering the adaptations to resistance exercise (Tesch et al. 2017; Martinez-Aranda and Fernandez-Gonzalo 2017). Peak power in the concentric and eccentric muscle actions in each repetition was recorded using encoder systems and associated software (squat; kMeter, Exxentric, knee extension; SmartCoach™, Stockholm, Sweden).

During sessions two, three and four, the aerobic bout, resistance bout, and control condition were performed. The interventions were randomized in a counterbalanced manner. The aerobic exercise bout consisted of 45 min of cycling at a target intensity (power output in Watts) equivalent to ~70% of *V̇*O_2max_ calculated using the linear relationship between *V̇*O_2_ and power output. Similar exercise intensities and protocols have been used by us and others to trigger acute systemic adaptations to aerobic exercise (Tvede et al. 1993; Strömberg et al. 2016). This protocol also represents an exercise routine commonly used to increase endurance capacity during training (Scribbans et al. 2016). The rate of perceived exertion and heart rate were measured every 5 min to adjust the workload such that a very strenuous effort was achieved. Lactate concentration was measured in venous blood before and after the aerobic exercise bout (Radiometer ABL 800 FLEX analyzer, Brønshøj, Denmark). The resistance exercise bout comprised 4 sets of 7 maximum squat repetitions on the flywheel ergometer followed by 4 sets of 7 maximum repetitions on a flywheel knee extension machine under strong verbal encouragement. Sets within an exercise were interspersed by a 3-min recovery, and a 5-min rest period was provided between exercises. In every set, two submaximal repetitions were used to initiate the movement and provide inertia to the systems. The type of exercise (i.e., isoinertial), number of exercises, repetitions, and sets used in this study are similar to previous reports showing significant gains in muscle mass and function with training (Fernandez-Gonzalo et al. 2014, 2016), and therefore represented a powerful activity to elicit local and systemic responses previously described for resistance exercise (Westcott 2012; Annibalini et al. 2019). During the control condition session, subjects rested for 45 min in a seated position in the laboratory facilities. Participants recorded their diet during the 24 h prior to the first session, and they replicated this diet in the following visits to the laboratory. The time of the day for each session was standardized for each participant (±1 h).

### Blood sampling and plasma preparation

A peripheral vein catheter was inserted in the cubital vein before sampling. Venous blood samples (40 ml) were collected using BD Vacutainer® EDTA immediately after and 30 min after the intervention. Blood samples were immediately centrifuged for 15 min at 2000 ×*g* using a refrigerated centrifuge. Plasma was then aliquoted and stored at −80 °C until further analysis.

### EV isolation

Extracellular vesicles were isolated from 5 ml plasma samples using Cushioned-Density Gradient Ultracentrifugation (C-DGUC) as previously described (Li et al. 2018) with minor modifications. Briefly, plasma samples were spun at 400 ×*g* for 10 min and then the supernatant was carefully collected before spinning again at 2000 ×*g* for 10 min. The supernatant was then collected and passed through a 0.22-micron filter. Dulbecco’s Phosphate buffered saline solution (DPBS) was added to the sample to bring the final volume up to 35 mL. Filtered samples were transferred to 38.5 mL ultracentrifuge tubes and underlaid with 2 mL 60% iodixanol using metal hub blunt 4-inch needles. Samples were then spun using a SW 32 Ti rotor in an Optima XPN 90 ultracentrifuge at 150,000 ×*g* for 3 h at 4℃. Upon completion of the run, 4-inch needles were used to collect 3 mL from the bottom of the ultracentrifuge tube. This was used to underlay a 12 mL ultracentrifuge tube that had a discontinuous gradient of 5, 10, and 20% iodixanol solution. Samples were then centrifuged in a SW 40 Ti rotor at 100,000 ×*g* for 18 h at 4 ℃ in an Optima XPN 90 ultracentrifuge.

Fractions 6 and 7 were collected at the end of the spin and were processed for downstream experiments. For lipidomics, samples were not concentrated, but for all other experiments, samples were concentrated to approximately 250 µL using an Amicon Ultra-4 Centrifugal Filter (Merck Millipore) by centrifugation at 4000 ×*g* for 30 min.

### EV quantification

EV samples were diluted in filtered phosphate-buffered saline (PBS) to achieve an appropriate concentration for detection and to mitigate particle aggregation. The NanoFCM NanoAnalyzer was utilized for data acquisition, which was conducted for a predetermined duration (60 s). Data were gated to exclude background noise and electronic artifacts by establishing appropriate thresholds based on the scatter profiles of calibration beads and a blank sample. EVs traversed the 488 nm laser and were identified by their characteristic light scatter profiles. A negative control (PBS alone) was analyzed to ensure that the observed scatter events were not attributable to buffer impurities or noise. Prior to sample collection, 250 nm Quality Control nanospheres were analyzed to verify proper laser alignment. These nanospheres, being of known concentration, were also employed to determine the NanoAnalyzer’s current flow rate, enabling sample concentration determination. Subsequently, a mixture of four different sizes of silica nanospheres (68, 91, 113, and 155 nm) was analyzed to generate a standard curve for determining the size of each sample population. The concentration of EVs in the sample was determined by quantifying the number of detected events (EVs) per unit volume, accounting for sample dilution. The NanoAnalyzer was operated using a low sample flow rate to minimize coincident events and optimize detection sensitivity.

### Transmission electron microscopy

Analysis of morphology of EVs was done as previously described by us (Valentino et al. 2021). Briefly, purified EVs were placed in a 30 µL droplet on parafilm and the surface of a carbon-formvar-coated copper grid was placed upside down to contact the samples for approximately 2 min. Afterward, the excess sample was wicked away by touching a piece of filter paper to the edge of the grid surface, followed by 30 s of air drying. The grid was placed upside down onto a droplet of stained solution (1% phosphotungstic acid) for approximately 2 min. Excess fluid was wicked from the grid following staining, leaving a thin aqueous film on the surface, which was left to air dry. Samples were examined at 80 kV under a Hitachi H7500 TEM.

### Western Blot

Samples for western blot (WB) analysis were concentrated as described above and were subsequently lysed with Pierce RIPA buffer (Thermo Scientific, #89,901) with Halt Phosphatase Inhibitor Cocktail (100×) (Thermo Scientific, #78426) and Halt Protease Inhibitor Cocktail (100×) (Thermo Scientific, #78429) on ice for 30 min. Protein concentration was determined by BCA (Bio-Rad, #5000113) and 10 μg protein was loaded per lane. Samples were loaded onto a gradient gel and ran at 250 V for 1 h, followed by transfer to a nitrocellulose membrane (Bio-Rad, #162-0146) using the Tans-blot Turbo (Bio-Rad). After transfer, the membrane was blocked with 5% Bovine Serum Albumin in TBST. The membranes were incubated overnight at 4 °C with primary antibodies (CD63, R&D Systems, #MAB50482, ApoE, Novus Biologicals, #NB110-6053155, Syntenin, Santa Cruz Biotechnology, #sc-515538). The next day, membranes were incubated with secondary antibodies (Bio-Rad, #Star117P and #Star121P) for 1 h at room temperature. Blots were developed by the ChemiDoc MP Imaging system (Bio-Rad).

### Lipidomics

EVs were isolated as described above and stored at −80 °C until they were shipped to Virginia Lipidomic and Metabolomics Center at the Virginia Commonwealth University. The extraction of lipids was conducted as follows: Samples were collected into 13 × 100 mm borosilicate tubes with a Teflon-lined cap (VWR, #60827-453). Lipids were released from tape strips by the addition of 2 mL of methanol and heated at 37 °C for 1 h. Tape backing was removed from the tube and 1 mL of chloroform was added along with the internal standard cocktail (10 uL). The contents were dispersed using an ultrasonicator at room temperature for 30 s. This single-phase mixture was incubated at 48 °C overnight. Debris were then pelleted in a centrifuge for 5 min at 5000 ×*g*, and the supernatant was transferred to a clean tube. The extract was reduced to dryness using a Speed Vac. The dried residue was reconstituted in 0.2 ml of the starting mobile phase solvent for untargeted analysis, sonicated for15 s, then centrifuged for 5 min in a tabletop centrifuge before transfer of the clear supernatant to the autoinjector vial for analysis.

The lipids were then separated by reverse phase LC using a Thermo Scientific Accucore Vanquish C18 + 2.1 (i.d.) × 150 mm column with 1.5 µm particles. The ultra-high performance liquid chromatography (UHPLC) used a binary solvent system at a flow rate of 0.26 mL/min with a column oven set to 55 °C. Prior to injection of the sample, the column was equilibrated for 2 min with a solvent mixture of 99% Mobile phase A1 (CH3CN/H2O, 50/50, v/v, with 5 mM ammonium formate and 0.1% formic acid) and 1% Mobile phase B1 (CH_3_CHOHCH_3_/CH_3_CN/H_2_O, 88/10/2, v/v/v, with 5 mM ammonium formate and 0.1% formic acid) and after sample injection (typically 10 mL), the A1/B1 ratio was maintained at 99/1 for 1.0 min, followed by a linear gradient to 35% B1 over 2.0 min, then a linear gradient to 60% B1 over 6 min, followed by a linear gradient to 100% B1 over 11 min, which held at 100% B1 for 5 min, followed by a 2.0 min gradient return to 99/1 A1/B1. The column was re-equilibrated with 99:1 A1/B1 for 2.0 min before the next run.

Each sample was injected two times for analysis in both positive and negative modes. For initial full scan MS (range 300–200 m/z) the resolution was set to 120,000 with a data-dependent MS2 triggered for any analyte reaching 3e6 or above signal. Data-dependent MS2 were collected at 30,000 resolutions. Data was analyzed using Thermo Scientific’s Lipid Search 4.2 software. Lipids were analyzed using LipiR package (Mohamed et al. 2020). After initial assessment of the data quality (visualization of the cross-molecule, -class, and -sample variability), samples were normalized using probabilistic quotient normalization (PQN) (Dieterle et al. 2006).

### Statistics

The concentration and size of EVs were analyzed using a repeated measures model, with concentration on a log scale. The model included fixed effects for sex, intervention (resistance exercise, aerobic exercise, and control), time, and the intervention by time interaction, with subject (nested within sex) as a random effect. An AR (1) correlation structure was used to model the repeated measures within subject and intervention. *F*-tests were used to test main effects and the intervention by time interaction. Mean comparisons were done using *t*-tests and Tukey adjusted *p*-values were used to account for the multiple comparisons. At each time point, Kruskal–Wallis tests were used to test for differences in percent abundance of lipids between interventions. Nemenyi’s non-parametric test with Tukey adjusted *p*-values was used when comparing pairs of interventions at each time point.

For lipidomic analysis, we utilized LipidR, which implements Limma (Ritchie et al. 2015), using multi-level experiments approach.

## Results

### Exercise results

The individual responses in terms of relative workload and heart rate from maximum levels, the Borg’s scale during the aerobic exercise bout, and the lactate values immediately after exercise (Table 2 and Supp. Figure 1) indicate that the intensity of this aerobic exercise session fell in the heavy domain (Black et al. 2017).These results suggest that the selected exercise session may have elicited the typical responses expected after aerobic exercise (Seidu et al. 2021). In relation to the resistance exercise bout, the participants of the study performed the resistance exercise with the appropriate technique, as indicated by the eccentric overload achieved in both the squat and knee extension exercises (Table 2 and Supp. Figure 2).

**Table 2:** Performance data from the aerobic exercise bout (AE) and the resistance exercise bout (RE) in the 10 participants that completed the study (5 men and 5 women)

	Workload AE (W)	HR AE (bpm)	RPE AE bout	Lactate pre AE (mmol/L)	Lactate post AE (mmol/L)
Mean	160.1	167.3	15.7	1.21	7.01
SD	41.0	10.2	0.5	0.58	2.12
Minimum	90.6	152.6	15.1	0.50	4.30
Maximum	216.3	182.5	16.4	2.45	10.75
	Concentric peak Power KE (W)	Eccentric peak Power KE (W)	Concentric peak Power squat (W)	Eccentric peak Power squat (W)	
Mean	182.9	226.2	538.3	559.3	
SD	101.8	110.3	237.6	206.2	
Minimum	92.3	125.5	199.0	262.7	
Maximum	384.2	455.6	949.5	906.5	

*RPE* Rate of perceived exertion, *HR* Heart rate, *KE* Knee extension

### EV characterization

**Fig. 1:**
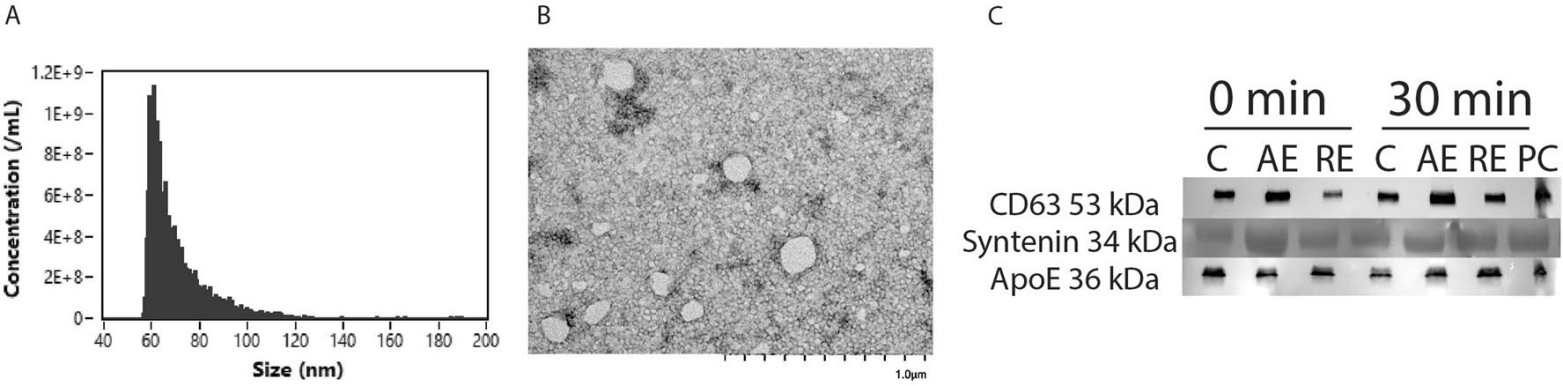
Extracellular vesicle characterization after exercise. Representative data depicting vesicle distribution (**A**), transmission electron microscopy image (**B**), and Western Blotting (**C**) of EV markers among control (C), aerobic (AE), and resistance (RE) exercise groups at both immediately post and 30 min following exercise. Plasma protein was used as positive control (PC)

We utilized cushioned-density gradient ultracentrifugation (C-DGUC) method as our isolation protocol to efficiently isolate small EVs from media and biofluids (Li et al. 2018; Duong et al. 2019). Following isolation, EVs were characterized in accordance with the guidelines set forth by the International Society of Extracellular Vesicles (ISEV) (Théry et al. 2018; Welsh et al. 2024). Nanoflow cytometry analysis (average vesicle size ranged between 60 and 160 nm) and visual confirmation with electron microscopy (Fig. 1A, B) confirmed the quality of the isolation method. Consistently, EV protein markers (CD63 and Syntenin) were present in our samples (Fig. 1C). We did, however, detect the expression of the lipoprotein marker, ApoE (Fig. 1C). Although the presence of ApoE has suggested contamination with HDL in the past, a recent study demonstrated a presence of a protein corona on the surface of plasma-derived EVs, with one of these proteins being ApoE (Tóth et al. 2021).

### Exercise does not induce changes in EV concentration or size

**Fig. 2:**
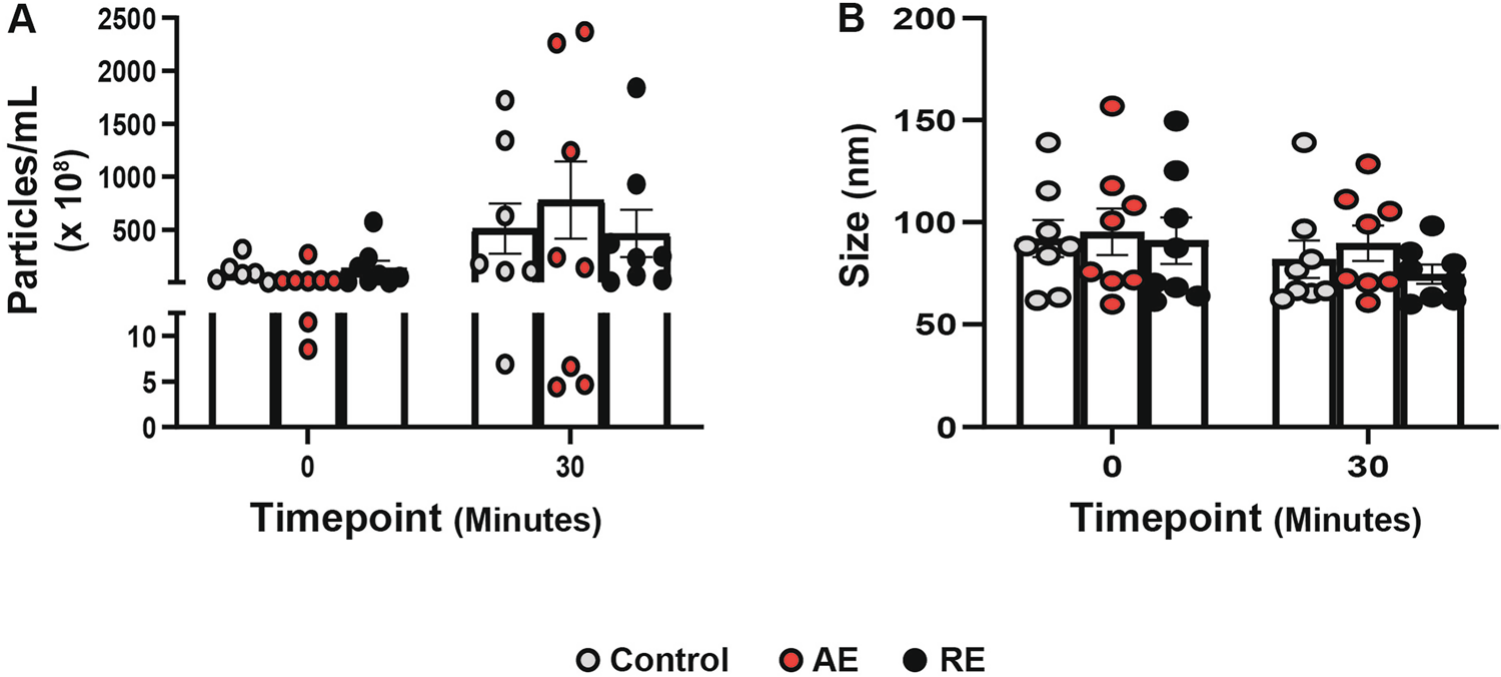
Exercise does not induced changes in EV concentration or size. (**A**) Concentration of particles (particles/mL × 10⁸) and (**B**) average size (nm) at 0 and 30 min relative to exercise (or rest for controls). Control (gray), aerobic exercise (AE; red), and resistance exercise (RE; black) groups are shown. Data are presented as mean ± SEM. No significant changes in particle concentration or size were observed post-exercise compared to control. Natural physiological variability in EV concentration was observed in all groups

Upon verifying the existence of small EVs in our isolates, we aimed to investigate if acute bouts of different types of exercise would impact the quantity and/or size of these vesicles. Our findings revealed that a bout of exercise did not lead to a substantial increase in EV concentration or cause changes in size compared to the control group, regardless of the type of exercise performed (Fig. 2A, B). Regarding time after the exercise bout, we observed a more uniform distribution of EVs immediately following the exercise as compared to 30 min after exercise. However, the overall number of particles varied considerably between individuals 30 min post-exercise, particularly in the aerobic exercise group (Fig. 2A). To quantify this variability, we calculated the coefficient of variation (CV) for particle concentration at each timepoint and for each group. The CV was notably higher at 30 min post-exercise, indicating increased inter-individual variability. The mean CV for the control group was 130.6%, for the AE group 132.0%, and for the RE group 135.4%. We also observed considerable variability in EV concentration across time points within the control group. This variation likely reflects natural physiological fluctuations unrelated to exercise, including factors such as circadian rhythm and hydration status.

### Exercise induces small changes in lipid content on EVs immediately after resistance but not aerobic exercise

**Fig. 3:**
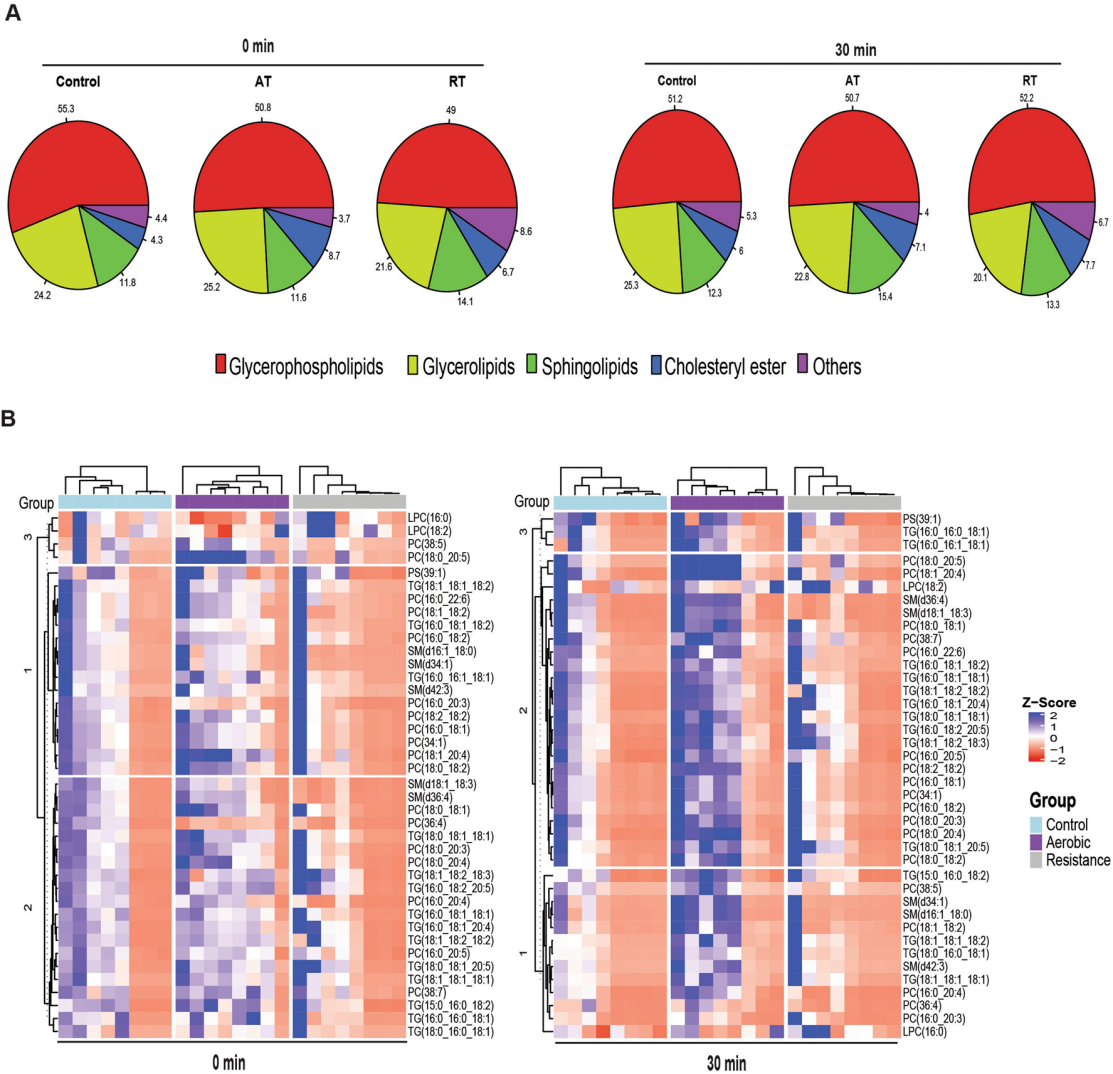
Changes in lipid content of EVs after resistance and aerobic exercise. Lipid classes (**A**) and species (**B**) of plasma-derived EVs among control (C), aerobic (AE), and resistance (RE) exercise presented as percent abundance or *Z*-score, respectively. The spread of *Z*-scores within groups highlights substantial inter-individual variability in lipid abundance

Next, we assessed the impact of different types of acute exercise on the lipid composition of EVs through untargeted lipidomics at different time points following the bout. Our analysis confirmed the presence of the most prevalent lipids found in EVs, including glycerophospholipids and sphingolipids (Fig. 3). Furthermore, our findings suggest that an acute exercise session, regardless of exercise modality, does not appear to cause significant alterations in the lipid composition of EVs (Fig. 3). However, some minor changes in the composition of major lipid classes were observed within EVs, mainly in the resistance exercise group compared to the control group (Fig. 3A). For instance, immediately after exercise, there was a decrease in glycerophospholipids (55% vs 49%; *P* < 0.05) in the resistance exercise group compared to the control group. Notably, these changes were not sustained 30 min after exercise (Fig. 3A).

In order to characterize the lipid composition of the EVs, we examined the top 40 most abundant lipid species immediately after and 30 min after an acute exercise session as compared to control. As demonstrated in Fig. 3B, we observed similar lipid species between groups and time points, supporting the absence of significant alterations in overall lipid class composition (Fig. 3A). However, closer inspection of individual lipid profiles reveals considerable variability among subjects, as evidenced by the broad range of *Z*-scores for specific lipid species within each group. This pattern indicates that, despite no group-level differences, individual responses to exercise or at rest varied markedly, likely reflecting differences in baseline metabolic status, exercise adaptation, or inter-individual biological variability.

**Fig. 4:**
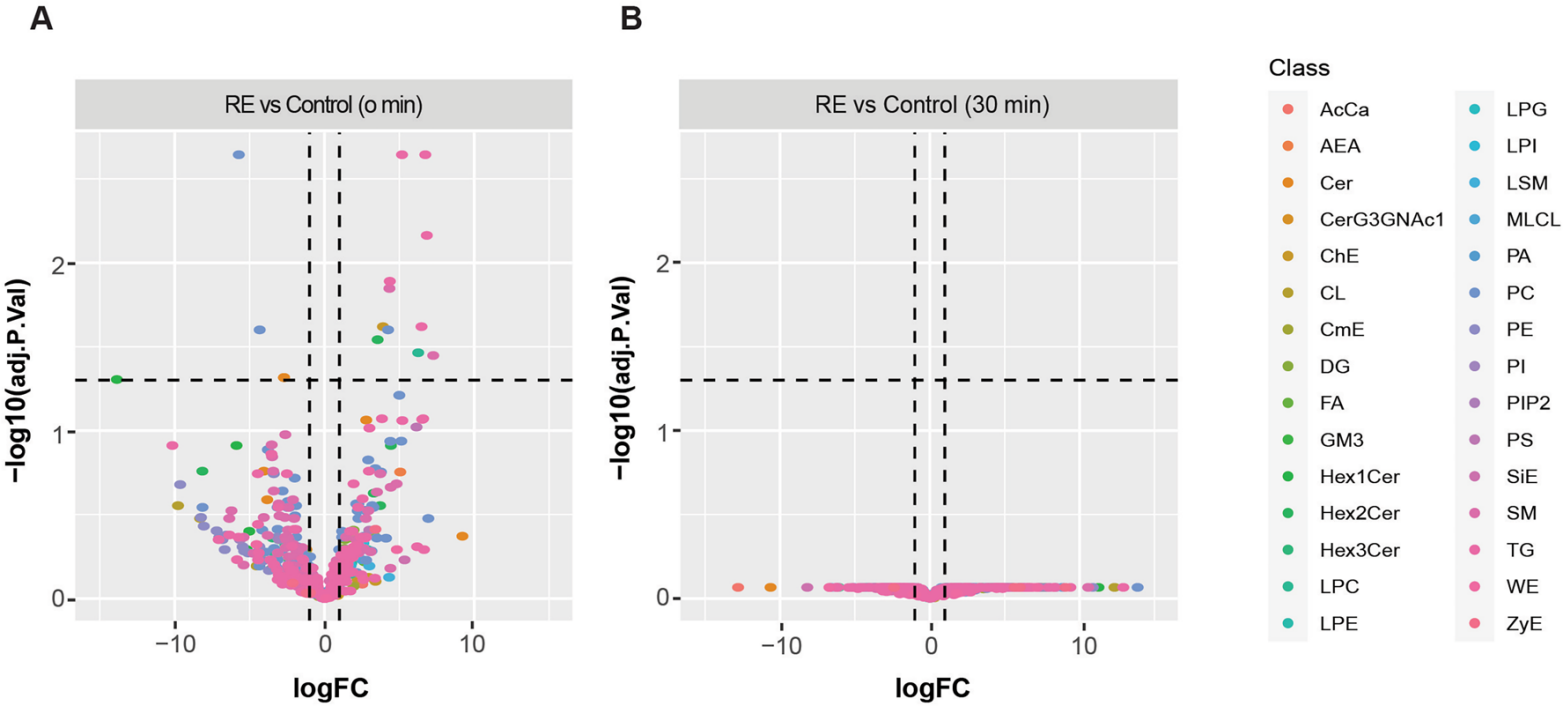
Lipid species comparisons between resistance exercise and control. Volcano plot depicting lipid species comparisons between resistance exercise (RE) and Control immediately post (**A**) and 30 min (**B**) following exercise

Finally, using a linear model with a multi-level experiment approach, we observed significant changes in the lipid species only when comparing the resistance exercise and control groups immediately post-exercise (Fig. 4A, B). Specifically, we observed a decrease in glycerophospholipids (PC 16:0/20:4 and PC 38:4) and an increase in cholesteryl ester (ChE) 18:3, sphingolipids (Hex2Cer t18:0/16:0 and SM d32:2), lyso-phosphatidylcholine (LPC) 16:1, and glycerolipids (TG 16:0/16:0/16:0 and TG 16:0/18:0/18:2). It is worth noting that we observed another eight differentially expressed lipids in the comparison between the resistance exercise and control groups (Fig. 4A). However, these lipids (TG 16:0/10:0/16:0, TG 16:0/10:0/18:3, TG 16:0/12:0/12:0, SM d17:1/18:3, PC 15:0/20:4, Cer d18:1/25:3, Cer t43:3, Hex1Cer d39:1) were of very low abundance and we consider them to be artificially elevated. No significant differences were observed between the aerobic and control or aerobic and resistance exercise groups (as shown in the Supp. Figure 3).

## Discussion

Extracellular vesicles have emerged as plausible mediators of intercellular communication and the systemic effects of exercise (Whitham et al. 2018). However, most studies examining the effects of exercise on EVs have focused on changes in miRNAs or proteins and have mostly involved aerobic exercise. As a result, there is limited information available on the contribution of resistance exercise to EV production, as well as the impact of exercise on the lipid content of EVs. Here, we employed a cross-over, randomized design involving both aerobic and resistance exercise to determine if different types of exercise result in changes in EV concentration, size, and lipid composition. Our results suggest that exercise, regardless of modality, does not change EV concentration or size. Furthermore, we found that resistance exercise, but not aerobic exercise, induced small changes in the lipid profile of circulating EVs. These results indicate that the lipid profile of EVs is not substantially affected during exercise and that resistance exercise, possibly due to a more eccentric type of stimulus, elicits changes in the lipid species.

Fruhbeis and colleagues were the first to present evidence suggesting that physical activity could regulate the concentration of EVs (Frühbeis et al. 2015). The group demonstrated that EVs are rapidly released into the circulation during aerobic exercise and return to pre-exercise levels within 90 min. Notably, their study also demonstrated that the kinetics of EV release were not identical between cycling and running exercises. The authors observed a rapid and considerable increase in EV number during cycling, while a moderate and sustained EV release was observed after running. These findings suggest that aerobic exercise can regulate EV release, with the mechanisms appearing to be dependent on the type of exercise. Although not investigated in this study, one possible explanation for these observed alterations could be attributed to muscle damage induced by the eccentric component of different types of exercise (i.e., running vs cycling). In fact, it has been demonstrated that eccentric exercise modalities affect EV-containing miRNA cargo when compared to concentric exercises (Banzet et al. 2013).

Despite these groundbreaking results, there is currently a lack of consensus in the field regarding the impact of exercise on EV concentration. For example, out of 16 studies that reported EV concentration following exercise in humans, nine studies indicated an increase in EV concentration post-exercise (Frühbeis et al. 2015; Helmig et al. 2015; Wilhelm et al. 2016; Bei et al. 2017; Whitham et al. 2018; Ma et al. 2018; Annibalini et al. 2019; Brahmer et al. 2019; Mohammad et al. 2021; McIlvenna et al. 2023), two studies showed a decrease (Eichner et al. 2020; Rigamonti et al. 2020), and five studies showed no change (Lovett et al. 2018; Hou et al. 2019; Nielsen et al. 2019; Just et al. 2020; Xiang et al. 2020). The disparity between these studies can likely be attributed to key differences in study design, including the type and intensity of exercise performed (e.g., endurance vs. resistance exercise), participant characteristics (e.g., fitness level, age, sex), timing of sample collection relative to exercise, and especially EV isolation and quantification techniques (e.g., ultracentrifugation, size exclusion chromatography, or precipitation-based methods). Variations in these factors may lead to differences in the purity, yield, and type of EVs analyzed, complicating direct comparisons across studies Additionally, natural physiological fluctuations in EV concentration, as observed in our control group, further highlight the dynamic nature of EV release even in the absence of exercise stimuli. Here, employing the cushioned-density gradient ultracentrifugation (C-DGUC) method (Li et al. 2018; Duong et al. 2019), which has been shown to effectively separate most non-EV fractions (Duong et al. 2019), we did not observe significant differences in EV size or concentration immediately following or 30 min after exercise compared to the control group, although our data trended toward an increased number of vesicles 30 min post exercise. Our findings align with several other studies (Lovett et al. 2018; Bertoldi et al. 2018; Hou et al. 2019; Nielsen et al. 2019; Just et al. 2020; Xiang et al. 2020; Vechetti et al. 2021) that also reported no changes, supporting the notion that when highly stringent EV isolation methods are used, exercise-induced changes in EV concentration may be more subtle.

Lipids are important components of EVs. The lipid composition of EVs influences their biogenesis, cargo sorting, interactions with target cells, and functional effects on recipient cells (Fyfe et al. 2023). Furthermore, lipids are not only constituents of cellular membranes but also key signaling mediators, acting as ‘bioactive lipids’ in several cellular processes, including immune regulation, inflammation, and maintenance of homeostasis (Chiurchiù and Maccarrone 2016). Indeed, it has been demonstrated that EVs carry bioactive lipids (e.g., eicosanoids) with the potential to contribute to a variety of biological functions, including modulation of distal immune responses (Boilard 2018). We sought to investigate the effects of different types of exercise on the lipid content of EVs. To our knowledge, there are no current studies that compare EV lipid profiles following both aerobic and resistance exercise. First, we confirmed that our global EV lipid profile identified a similar lipid makeup as previous studies (Record et al. 2014; Haraszti et al. 2016; Skotland et al. 2020), with the most abundant lipids being phosphatidylcholine (PC), phosphatidylserine (PS), sphingomyelin (SM), and lyso-phosphatidylcholine (LPC). In addition, we determined that eight lipids were significantly altered in resistance exercise compared to control immediately post-exercise. Surprisingly, we did not observe any changes following aerobic exercise.

While we have not investigated the mechanisms associated with the differences between aerobic and resistance exercise in terms of EV lipid composition, we hypothesize that the changes observed in our study were attributed to the stimulating and/or damaging effects promoted by resistance exercise. This is in line with previous reports indicating slight increases in muscle damage markers after isoinertial resistance exercise in both men and women (Fernandez-Gonzalo et al. 2014). In fact, in our study, the majority of the lipid species altered in EVs following resistance exercise were related to inflammatory responses. For example, we observed a decrease in PC 16:0/20:4 and PC 38:4, glycerophospholipids that play a significant role in membrane structure and function (Gibellini and Smith 2010), with some species containing arachidonic acid (AA) (Takamori et al. 2006). Arachidonic acid-containing PC can be hydrolyzed by phospholipase A2 (PLA2) into AA, which in turn can be metabolized into eicosanoids such as prostaglandins and leukotrienes and exert pro-inflammatory cytokine responses (Murakami and Kudo 2002). While speculative, the decrease in these PC species could indicate a reduced pro-inflammatory potential of EVs after resistance exercise, reflecting the broader anti-inflammatory effect often observed post-exercise, contributing to the resolution of inflammation and promotion of recovery. Alternatively, after resistance exercise, the body may increase the demand for these mediators to manage inflammation, muscle repair, and recovery. This could result in the depletion of PC species containing arachidonic acid as it is mobilized for these purposes, leading to their reduced presence in EVs. Further investigations are required to determine whether the decrease of PC16:0/20:4 and PC 38:4 is a result of a flux of conversion to arachidonic acid and downstream metabolites.

We also observed an increase in ChE18:3, a polyunsaturated fatty acid, specifically linolenic acid, esterified to cholesterol. While there is limited information regarding the role of ChE18:3, polyunsaturated fatty acids, which includes linolenic acid, have anti-inflammatory properties (Calder 2005). Thus, an increase in ChE18:3 could indicate that these vesicles are involved in modulating inflammation post-exercise, perhaps by delivering anti-inflammatory lipids to target cells.

LPC, a phospholipid, has been found to increase with disease states such as diabetes, renal failure, and cardiovascular conditions (Rabini et al. 1994; Wong et al. 1998; Sasagawa et al. 1998). In addition, LPC has been demonstrated to be pro-inflammatory lipid mediators generated from PLA2-catalyzed hydrolysis of PC, the fundamental component of cell membranes (Hung et al. 2012). Exercise is known to induce a transient inflammatory response, which is typically followed by an anti-inflammatory effect as part of the recovery process. This controlled inflammation is essential for tissue repair and adaptation. Although speculative, the increase in LPC 16:1 on EVs may potentially play a role in this process by modulating immune cell activity and cytokine production, thereby contributing to the balance between pro- and anti-inflammatory signals. We did also observe an increase in sphingolipids, Hex2Cer t18:0/16:0 and SM d32:2, which have been demonstrated to play critical roles in modulating membrane fluidity and inflammatory responses (Maceyka and Spiegel 2014; Varela et al. 2024). The increase in all these species observed in EVs following resistance exercise suggests that exercise stimulates EV formation that could potentially impact inflammation.

Finally, we also observed an increase in triglycerides. While this could be indicative of the presence of contaminants in the samples, it has been demonstrated that EVs also contain triglycerides (Glover et al. 2019). The increase in triglycerides in EVs could also represent an increase in lipolysis following resistance exercise. Indeed, we have demonstrated that a hypertrophic stimulus induced lipolysis in white adipose tissue, and this adaptation was mediated by EVs (Vechetti et al. 2021).

When interpreting the results presented in this study, there are a few considerations to bear in mind. First, using a percentage of *V̇*O_2max_ to determine the intensity of the aerobic exercise bout can increase inter-individual differences in exercise intensity (Lansley et al. 2011). However, given the physiological readouts during and after the bout, we believe the exercise protocol used here served to trigger the systemic responses associated with intense aerobic exercise. Secondly, we examined whole body plasma, which contains EVs from multiple cell types. Our hypothesis posited that exercise would alter the lipid composition and modify the release and seize of EVs, which we observed only marginally following resistance exercise. It is conceivable that analyzing plasma was too broad of an approach, as we do not know the precise origin of these EVs, nor their role in specific target tissues. This is particularly relevant for skeletal muscle-derived EVs, as it has been demonstrated that skeletal muscle accounts for 1–5% of plasma EVs (Guescini et al. 2015). We also observed ApoE positive particles, indicating the presence of HDL. While this most likely suggests contamination with lipoproteins, a recent study has shown that the presence of lipoprotein in EVs derived from plasma could be part of a protein corona (Tóth et al. 2021). Finally, although we included men and women in the study, the low number of participants of each sex prevented a sex-specific analysis, so future studies should specifically address this question.

In summary, our findings indicate that different forms of exercise do not alter EV concentration and have only minor effects on EV lipid composition. Despite the prevailing hypothesis that skeletal muscle functions as a secretory organ during and after exercise to mediate the positive systemic effects of physical activity, our data suggest that the EV-containing lipids are not the major mechanism utilized by the cells to mediate exercise-induced adaptations. It is possible that EVs released by skeletal muscle are either dispersed into the systemic circulation or absorbed by adjacent cells, which would complicate the identification of changes in muscle-secreted EVs in plasma. Alternatively, skeletal muscle may not be the primary organ responsible for driving exercise-induced adaptations. Finally, although descriptive, our study provides important information regarding the lipid composition after a bout of both aerobic and resistance exercise.

## Supplementary Information

Below is the link to the electronic supplementary material.

Supplementary file1 (DOCX 18 KB)

Supplementary file2 (JPG 686 KB)

Supplementary file3 (PDF 44 KB)

Supplementary file4 (PDF 36 KB)

## Data Availability

Data are available from the corresponding authors on reasonable request.

## Abbreviations

AA: Arachidonic acid
C-DGUC: Cushioned-density gradient ultracentrifugation
ChE: Cholesteryl ester (ChE)
CON: Control, resting condition
EV: Extracellular vesicle
HDL: High-density lipoprotein
HR_max_: Maximum heart rate
ISEV: International society of extracellular vesicles
LPC: Lyso-phosphatidylcholine
MVIC: Maximal voluntary isometric contraction
PBS: Phosphate-buffered saline
PC: Phosphatidylcholine
PLA2: Phospholipase A2
PS: Phosphatidylserine
PUFAs: Polyunsaturated fatty acids
RER: Respiratory exchange ratio
SM: Sphingomyelin
TEM: Transmission electron microscopy
*V̇*O_2max_: Maximal oxygen uptake
*W*_max_: Maximal workload
