# Supplementary material for: The effects of aerobic and resistance exercise on the lipid profile of extracellular vesicles
Source: Eur J Appl Physiol. 2025 Oct 1;126(3):1573–87. doi: 10.1007/s00421-025-05973-1 (PMC13013174; doi:10.1007/s00421-025-05973-1)
Supplement: Supplementary file 1 — Supplementary file1 (DOCX 18 KB) [file 421_2025_5973_MOESM1_ESM.docx]

**Supplementary Figure 1. Individual response to the aerobic exercise bout.** Relative workload (%; left pane) and heart rate (%, middle pane) from maximum levels achieved during the maximal oxygen uptake, and Borg’s scale values (right panel) during the 45-minute aerobic exercise bout. Each color represents a particular participant. The same participant is shown with the same color in all the graphs.

**Supplementary Figure 2. Individual performance for the resistance exercise bout.** Power (Watts) achieve during the knee-extension (upper panel) and squat (lower panel) exercise for concentric (green) and eccentric (purple) muscle action across all the sets performed. Each dotted color represents the values of a particular participant. The same participant is shown with the same color in all the graphs.

**Supplementary Figure 3.** **Lipid specie comparisons among Control, Aerobic, and Resistance exercise.** Volcano plot depicting lipid specie comparisons among Control, Aerobic and Resistance exercise immediately post and 30 minutes following exercise.
