# Supplementary figures and images for: The effects of aerobic and resistance exercise on the lipid profile of extracellular vesicles
Source: Eur J Appl Physiol. 2025 Oct 1;126(3):1573–87. doi: 10.1007/s00421-025-05973-1 (PMC13013174; doi:10.1007/s00421-025-05973-1)

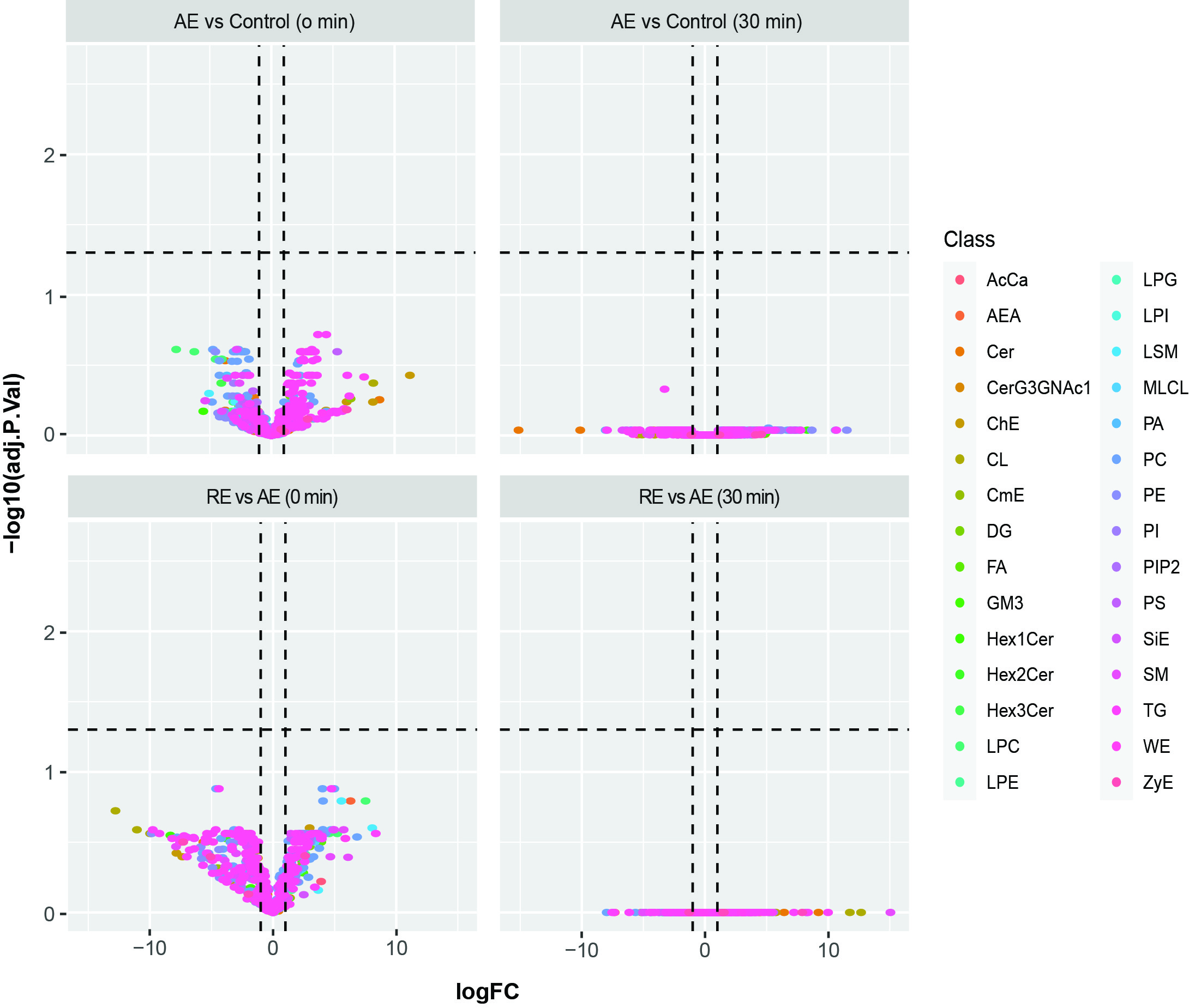

Supplement: Supplementary file 2 — Supplementary file2 (JPG 686 KB) [file 421_2025_5973_MOESM2_ESM.jpg]

## Knee extension power

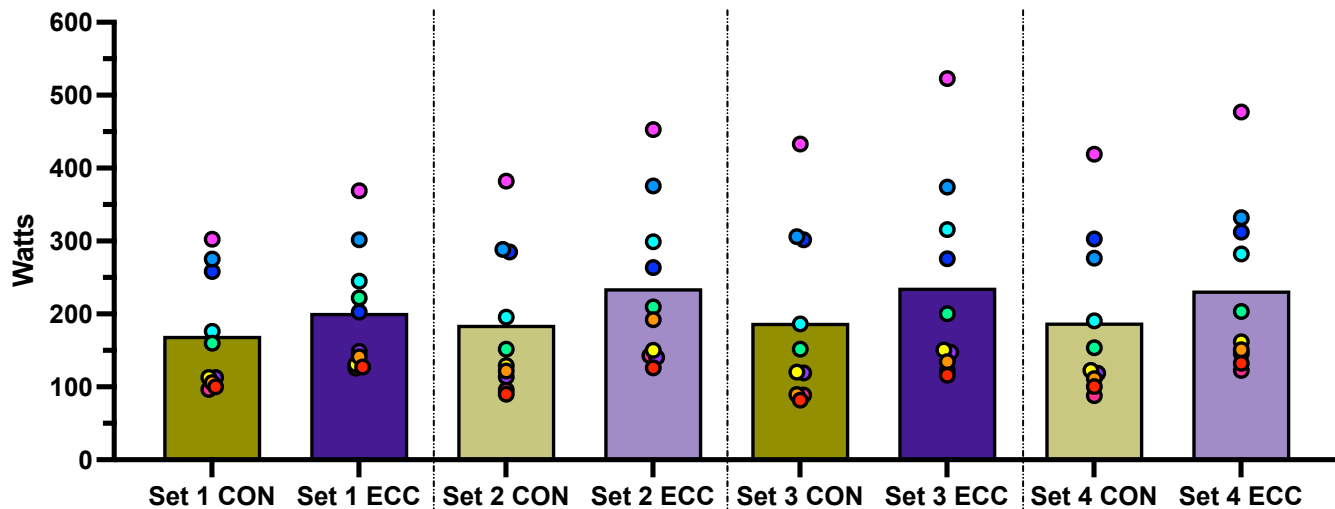

## Squat power

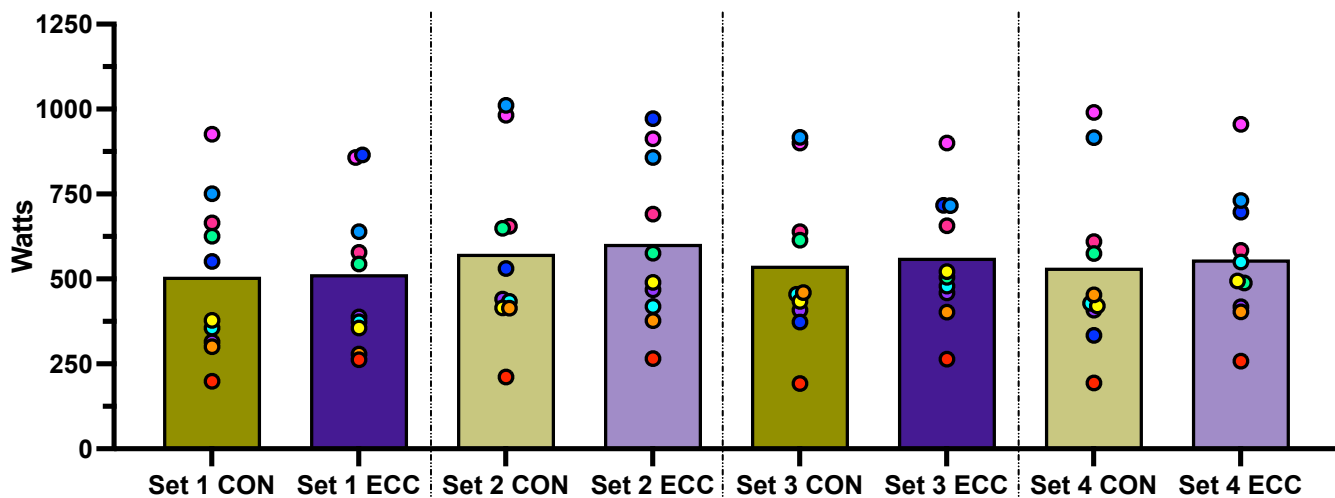

Supplement: Supplementary file 3 — Supplementary file3 (PDF 44 KB) [file 421_2025_5973_MOESM3_ESM.pdf]
