# Supplementary material for: The effects of aerobic and resistance exercise on the lipid profile of extracellular vesicles
Source: Eur J Appl Physiol. 2025 Oct 1;126(3):1573–87. doi: 10.1007/s00421-025-05973-1 (PMC13013174; doi:10.1007/s00421-025-05973-1)
Supplement: Supplementary file 4 — Supplementary file4 (PDF 36 KB) [file 421_2025_5973_MOESM4_ESM.pdf]

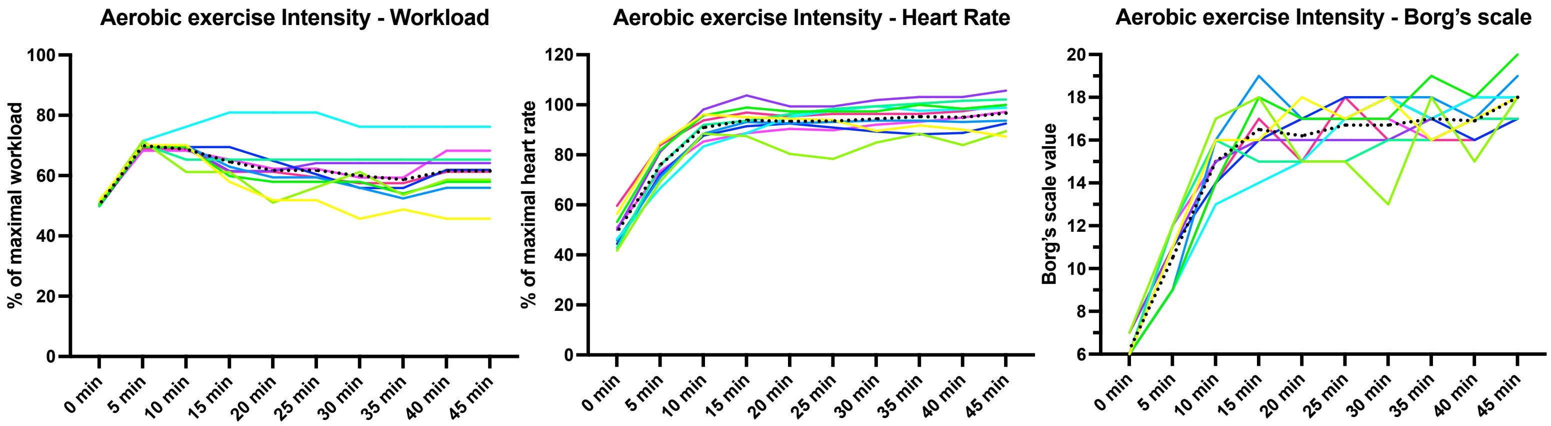

Aerobic exercise Intensity - Workload
% of maximal workload
0
20
40
60
80
100
0 min
5 min
10 min
15 min
20 min
25 min
30 min
35 min
40 min
45 min
Aerobic exercise Intensity - Heart Rate
% of maximal heart rate
0
20
40
60
80
100
120
0 min
5 min
10 min
15 min
20 min
25 min
30 min
35 min
40 min
45 min
Aerobic exercise Intensity - Borg's scale
Borg's scale value
6
8
10
12
14
16
18
20
0 min
5 min
10 min
15 min
20 min
25 min
30 min
35 min
40 min
45 min
